# Variation in Cell Signaling Protein Expression May Introduce Sampling Bias in Primary Epithelial Ovarian Cancer

**DOI:** 10.1371/journal.pone.0077825

**Published:** 2013-10-28

**Authors:** Gabriele Mittermeyer, Katharina Malinowsky, Christian Beese, Heinz Höfler, Barbara Schmalfeldt, Karl-Friedrich Becker, Stefanie Avril

**Affiliations:** 1 Department of Pathology, Technische Universität München, Munich, Germany; 2 Institute of Pathology, Helmholtz Zentrum München, Neuherberg, Germany; 3 Department of Obstetrics and Gynecology, Technische Universität München, Munich, Germany; Rajiv Gandhi Centre for Biotechnology, India

## Abstract

Although the expression of cell signaling proteins is used as prognostic and predictive biomarker, variability of protein levels within tumors is not well studied. We assessed intratumoral heterogeneity of protein expression within primary ovarian cancer. Full-length proteins were extracted from 88 formalin-fixed and paraffin-embedded tissue samples of 13 primary high-grade serous ovarian carcinomas with 5–9 samples each. In addition, 14 samples of normal fallopian tube epithelium served as reference. Quantitative reverse phase protein arrays were used to analyze the expression of 36 cell signaling proteins including HER2, EGFR, PI3K/Akt, and angiogenic pathways as well as 15 activated (phosphorylated) proteins. We found considerable intratumoral heterogeneity in the expression of proteins with a mean coefficient of variation of 25% (range 17–53%). The extent of intratumoral heterogeneity differed between proteins (p<0.005). Interestingly, there were no significant differences in the extent of heterogeneity between phosphorylated and non-phosphorylated proteins. In comparison, we assessed the variation of protein levels amongst tumors from different patients, which revealed a similar mean coefficient of variation of 21% (range 12–48%). Based on hierarchical clustering, samples from the same patient clustered more closely together compared to samples from different patients. However, a clear separation of tumor versus normal tissue by clustering was only achieved when mean expression values of all individual samples per tumor were analyzed. While differential expression of some proteins was detected independently of the sampling method used, the majority of proteins only demonstrated differential expression when mean expression values of multiple samples per tumor were analyzed. Our data indicate that assessment of established and novel cell signaling proteins as diagnostic or prognostic markers may require sampling of serous ovarian cancers at several distinct locations to avoid sampling bias.

## Introduction

Ovarian cancer is the second most common gynecological malignancy and the one with the highest mortality in the Western world [1]. During early stages the disease is mostly asymptomatic and consequently most patients are diagnosed with advanced stages resulting in a poor five-year overall survival rate of less than 40% [2]. Standard treatment for patients with ovarian cancer is surgery followed by platinum and taxane based combination chemotherapy. Although most patients show initial response to chemotherapy, the majority subsequently develops resistance and 50–75% of patients relapse within five years [3]. The assessment of cell signaling proteins offers the opportunity to identify potential new drug targets as well as to predict response to treatment and aid in individualized treatment decisions [4]. In order to serve as a biomarker for an optimized targeted therapy approach the identification and quantitative analysis of target structures and/or respective downstream signaling cascades is of high relevance. However, potential intratumoral heterogeneity of cell signaling protein expression may introduce a sampling bias and has not been comprehensively assessed in ovarian cancer.

A number of cell signaling proteins have been previously identified as possible therapeutic targets or as potential prognostic or predictive biomarkers. These include VEGF, VEGFR, PDGFR, EGFR or HER2, PI3K, Akt, mTOR and others [5], [6], [7], [8]. Similarly important is the activation of cell signaling pathways reflected by the phosphorylated forms of the target proteins or components of their respective downstream signaling pathways [9], [10], [11].

The overall goal of this study was to assess the level of heterogeneity of cell signaling protein expression in serous ovarian cancer. We analyzed 36 cell signaling proteins, including 15 phosphorylated proteins representing proliferation and angiogenesis related pathways. We additionally investigated the potential impact of heterogeneity on detection of differential protein expression between tumor and normal tissue or between tumor subgroups.

In comparison, we assessed the physiological variation of protein expression in normal serous epithelial tissue between different patients. Fallopian tube epithelium from healthy individuals and from uninvolved contralateral tubes of cancer patients was utilized as a reference tissue, since previous studies have demonstrated that serous cancers are molecularly most closely related to tubal epithelium. Fallopian tube epithelial cells have been suggested as the cells of origin for at least some high-grade serous carcinomas [12], [13].

For the analysis of large numbers of samples and target proteins as applied in this study, conventional immunoblot methodology is not suitable as one would need more than 3500 Western blot lanes to conduct a single analysis of all samples and antibodies. The reverse phase protein array (RPPA) allows the simultaneous analysis of multiple samples for the expression of several proteins under the same experimental conditions [14], [15]. Analysis of proteins in duplicates and serial dilutions enables reproducible quantitative detection of protein expression. RPPA has widely demonstrated its feasibility for the analysis of cryo-preserved clinical samples [16], [17], [18]. More recently, our group and others established that RPPA technology also reliably allows the analysis of formalin-fixed and paraffin-embedded (FFPE) tissue samples [19], [20], [21], [22] and is an adequate tool to address protein heterogeneity within such samples [23].

## Materials and Methods

### Tissue Samples

**Figure 1:**
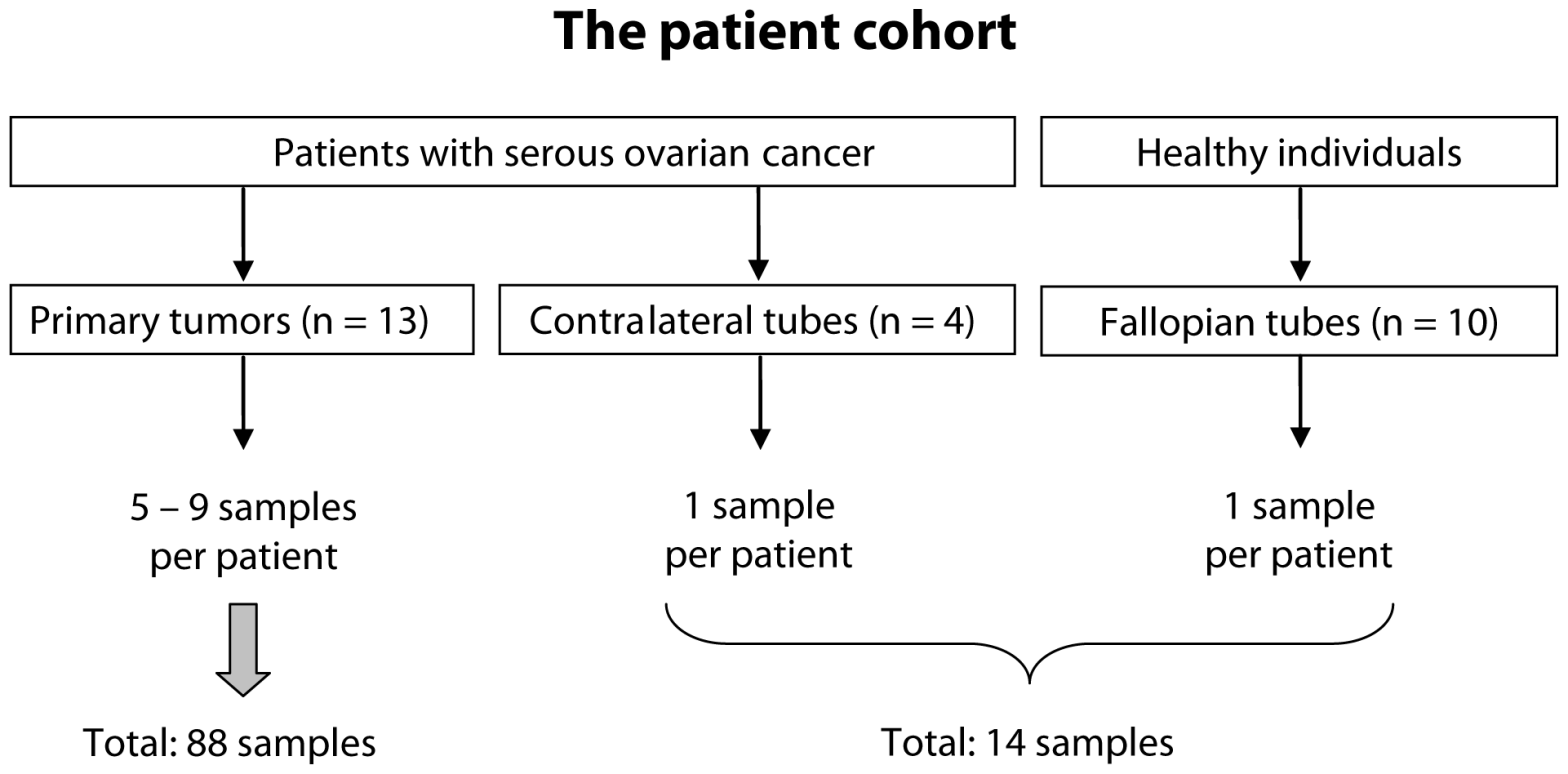
Schematic illustration of patient cohort and sample selection.

A total of 88 FFPE samples from 13 patients with primary high-grade serous ovarian cancer were studied. 14 samples of normal fallopian tube epithelium, including 10 healthy individuals and 4 uninvolved contralateral tubes from cancer patients were used as reference. A schematic of the patient cohort and selection of samples is depicted in Figure 1.

For all 13 cases, 5–9 tissue samples were available. In ten patients with bilateral tumors, samples were obtained from both ovaries.

H&E stained sections of all paraffin embedded samples were reviewed by an experienced pathologist (S.A.) to characterize the histological subtype, percentage of viable invasive tumor cells, fibrosis or necrosis, percentage of infiltrating inflammatory cells, and frequency of mitoses.

### Protein Extraction

All tissue samples from the same patient were processed simultaneously. Protein extraction was performed as previously described [24]. Briefly, FFPE tissue sections were deparaffinized, and proteins were extracted using EXB Plus buffer and heat treatment (Qiagen, Hilden, Germany). 2–7 sections of 10 µm thickness were processed in 100–170 µl of extraction buffer. The Bradford protein assay (BioRad, Hercules, US) was used according to the manufacturer’s instructions to determine protein concentrations. Protein concentrations were adjusted to 2 mg/ml with EXB Plus buffer. A Western blot probing for β-actin was performed from randomly selected lysates (n = 11) to demonstrate successful protein extraction and suitability for reverse phase protein array analysis. All protein lysates produced a clear β-actin band on the Western blot.

### Analysis of Protein Expression by Reverse Phase Protein Arrays (RPPA)

The expression levels of 36 proteins including 15 phosphorylated proteins were determined by RPPA. Antibodies and experimental conditions are summarized in **Table S1**. RPPAs were generated using the SpotBot® Extreme Microarray Spotter according to the manufacturer’s instructions (Arrayit, Sunnyvale, CA 94089, USA). For every lysate and dilution (adjusted (2 mg/ml), 1∶2, 1∶4, 1∶8, 1∶16, extraction buffer), 2 replicates were applied onto a nitrocellulose coated glass slide (Grace Bio-Labs, Bend, US), which produced 12 data points per sample.

Immunodetection was performed similar to Western blot analysis and as previously described [25]. For estimation of total protein amounts, arrays were stained in parallel with Sypro Ruby Protein Blot Stain (Molecular Probes, Eugene, USA) according to the manufacturer’s instructions. Further details of the RPPA methodology, validation, and technical reproducibility have been previously described [23], [25]. All antibodies used in this study were validated for specificity by Western blot analysis.

### Statistical Analysis

Intratumoral heterogeneity as well as the range of protein expression amongst different patients (inter-patient variation), and the variability of protein expression between fallopian epithelium from healthy individuals were assessed using the *coefficient of variation* (CV). The CV, defined as the ratio of the standard deviation to the mean multiplied by 100, provides a relative measure for variation independent of the absolute values, and therefore allows comparing the variation of proteins with different absolute expression levels.

Intratumoral heterogeneity was assessed separately for each protein by calculating the CV of all primary tumor samples from the same patient. As summary statistic, the root-mean-square (RMS) average of the CVs [26] was calculated to include all 13 patients to assess the overall intratumoral heterogeneity for a given protein.

The variation among tumors from different patients was assessed for each individual protein by calculating the CV of mean expression values among the different patients. Results are displayed graphically using box-plots showing the median expression value, 25^th^ and 75^th^ quartiles, and whiskers (1.5 times the interquartile range) for each patient.

The Friedman test was used to compare CVs for different proteins and the Mann Whitney test was used to compare protein expression between unrelated sample groups at a two-sided 5% level of significance. All statistical analyzes were performed using IBM Statistics (IBM Corporation, Version 19.0).

To compare protein expression between normal and tumor tissues and to visualize variation between samples from individual patients, non-supervised hierarchical clustering was performed by using Cluster and TreeView software [27]. Following log transformation and centering to median values, average hierarchical clustering was performed for the calculation of Spearman rank correlation.

## Results

### Morphological Assessment of Tissue Samples

All samples showed a tumor cellularity of >70% and <10% inflammatory cells. There were no significant differences in epithelial tumor cell content and tumor stroma or inflammatory cell infiltrates between samples from the same patient. In addition, no regional differences were identified in mitotic activity between samples from the same patient.

### Intratumoral Heterogeneity of Protein Levels

**Figure 2:**
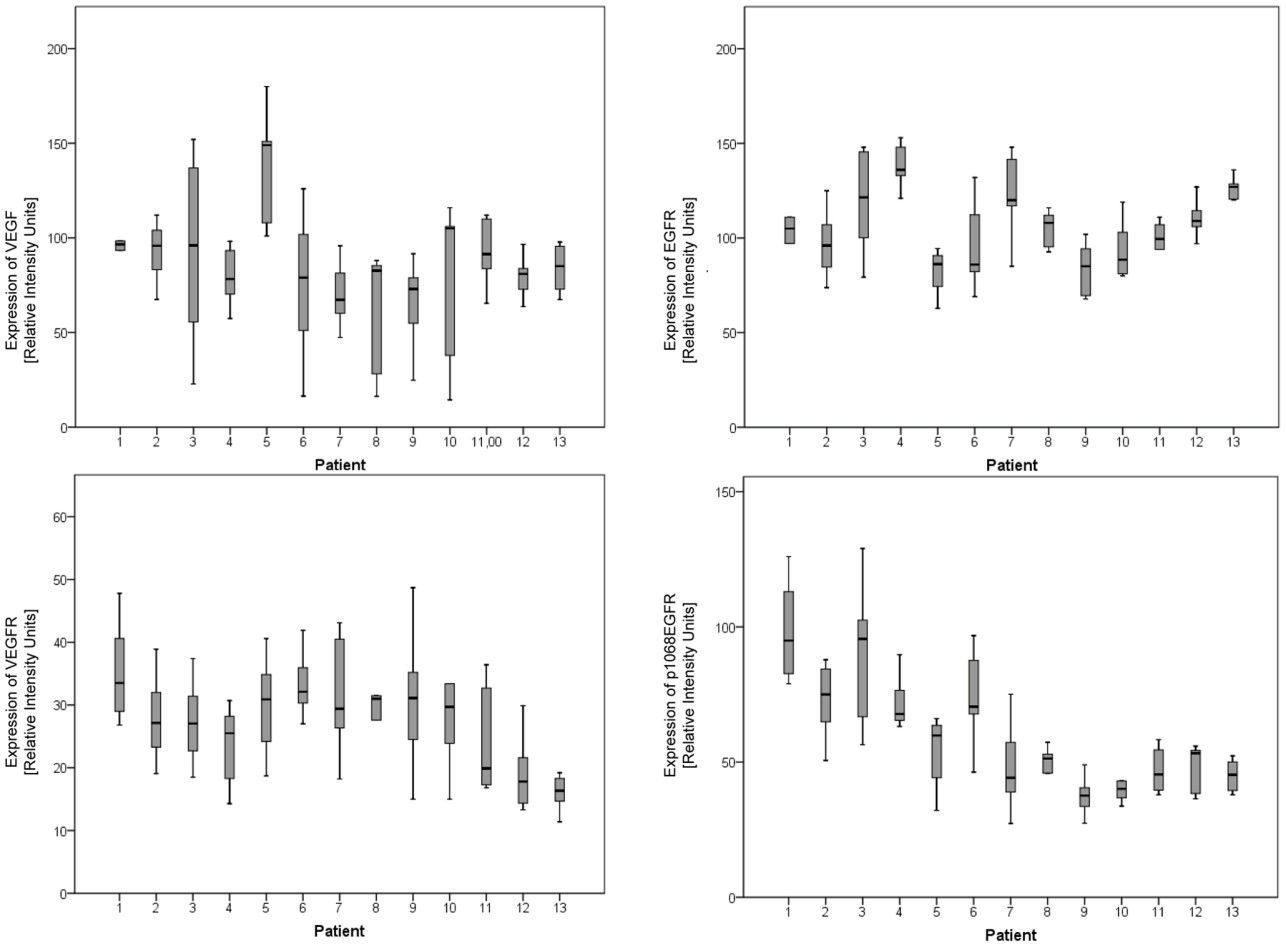
Intratumoral heterogeneity and variation among different patients for the expression of 4 exemplary proteins (VEGF, VEGFR, EGFR and p1068EGFR) assessed by reverse phase protein arrays. Box plots show the median (line within the box), 25th and 75th percentiles, and whiskers are showing 1.5 times the interquartile range.

All 36 proteins analyzed in this study showed considerable intratumoral heterogeneity with a mean CV of 25% (range 17–53%) (Table 1 and Table S2). The extent of intratumoral heterogeneity differed among the 36 proteins analyzed (p = 0.005), with p4EBP1, pp44/42 MAPK and VEGF showing highest variability, and EGFR, JNK/SAPK and p38MPK showing least variability within individual tumors. There was no significant difference in the extent of intratumoral heterogeneity between phosphorylated (mean CV 28%) and non-phosphorylated (mean CV 23%) proteins (p = 0.252). The intratumoral heterogeneity of all proteins is summarized in Table S2. Figure 2 illustrates intratumoral heterogeneity for the exemplary proteins VEGF, VEGFR p1068EGFR and EGFR.

**Table 1:** Comparison of intratumoral heterogeneity and variation among patients for all proteins, phosphorylated and non-phosphorylated proteins.

	Intratumoral mean CV (range) [%]			Inter-patient mean CV (range) [%]		
	Overall	Phospho	Non-phospho	Overall	Phospho	Non-phospho
**Tumor**	25 (17–53)	28 (21–53)	23 (17–36)	21 (12–48)	25 (14–48)	18 (12–23)
**Healthy tubes**	–	–	–	27 (14–47)	29 (17–47)	25 (14–44)
**Contralateral tubes**	–	–	–	31 (3–78)	36 (15–78)	26 (3–45)

CV, coefficient of variation.

Overall, all proteins combined; Phospho, all phosphorylated proteins combined; Non-phospho, all non-phosphorylated proteins combined.

Healthy tubes, fallopian tube epithelium from healthy individuals.

Contralateral tubes, morphologically normal contralateral fallopian tube epithelium from ovarian cancer patients.

### Variation of Protein Levels Among Patients

The variation of protein expression among different patients was similar to the extent of intratumoral heterogeneity with a mean CV of 21% (range 12–48%). The inter-patient variability differed significantly among the proteins analyzed. While Hif1α, PDGF and PI3K demonstrated low variation between all patients analyzed, p4EBP1, pp44/42 MAPK and p1068EGFR showed a very high inter-patient variability. Variation between different patients was not significantly different for phosphorylated (mean CV 25%) and non-phosphorylated (mean CV 18%) proteins (p = 0.072). Table S2 summarizes the variation among different patients for all proteins. Figure 2 illustrates the variation among patients for the exemplary proteins VEGF, VEGFR, EGFR and p1068EGFR.

Inter-patient variation of protein expression was also assessed for normal serous epithelium of fallopian tubes from healthy individuals and for morphologically normal contralateral tubes of cancer patients. The inter-patient variability of tubal epithelium from healthy individuals (mean CV 27%, range 14–47%) was similar to inter-patient variation of contralateral tubes from cancer patients (mean CV 31%, range 3–78%). Overall, variation of normal serous epithelium among different individuals was in a similar range compared to variation between tumors from different patients (21%). Variability was not different between phosphorylated and non-phosphorylated proteins in either type of reference tissue. Results are summarized in Table 1.

### Variation between Individual Tumor Samples Assessed by Hierarchical Clustering

**Figure 3:**
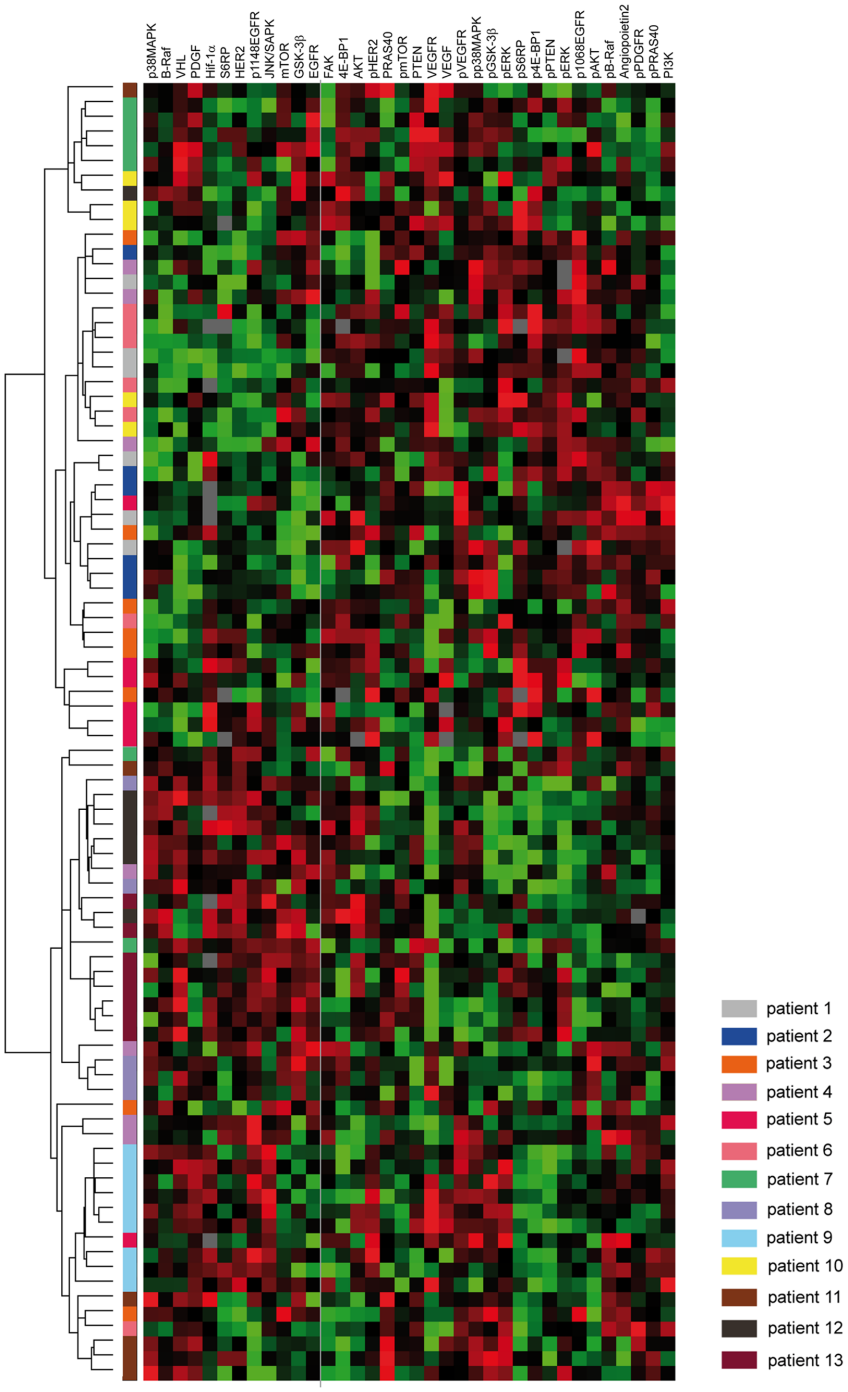
Variation between 88 individual tumor samples from 13 patients assessed by non-supervised hierarchical clustering based on expression of 36 proteins. Different patients are color-coded as indicated in the figure legend. High relative expression of proteins is shown in red and low expression in green color. Grey spaces indicate missing data points.

To assess variation between individual tumor samples we performed non-supervised hierarchical clustering of all 88 individually taken tumor samples from 13 patients based on the expression of all 36 proteins analyzed (**Figure 3**). Samples from the same patient clustered more closely together compared to samples from different patients. Nevertheless, the samples of four patients (patients 3, 4, 6, 11) were scattered throughout all clusters, and for the remaining patients two or more samples per patient did not cluster with the rest. There was no patient for whom all samples clustered together (**Figure 3**).

### Relevance of Heterogeneity for Distinction between Tumor Versus Normal Tissue

#### Clustering of tumor versus normal tissue

We assessed the influence of heterogeneity on molecular classification of distinct tissue samples as cancerous or normal based on hierarchical clustering. There was no clear separation of tumor samples versus normal serous epithelium when using all individual tumor samples per case (data not shown). In contrast, analysis of mean expression values for each tumor resulted in very good separation of normal and tumor tissues (**Figure S1**).

#### Differential protein expression in tumor versus normal tissue

We next assessed differences in protein expression between ovarian cancer and normal serous epithelium by either taking randomly selected single samples per case or mean protein expression values. The analysis was repeated three times with three different randomly selected single samples per case and results were compared to those obtained using the mean expression values per case as reference standard.

Sixteen proteins identified as differentially expressed using mean expression values of all samples per tumor (AKT, pAKT, Angiopoietin 2, B-Raf, p1068EGFR, p1148EGFR, FAK, pGSK-3β, pGSK-3β, Hif-1α, JNK/SAPK, mTOR, pmTOR, p38 MAPK, pPRAS40, PRAS40, PTEN, pPDGFR, S6RP) were not detected when analyzing randomly selected single samples per case. Ten proteins, namely pB-Raf, EGFR, HER2, 4E-BP1, pPTEN, pVEGFR, VEGF, VEGFR, pS6RP, and VHL were identified as differentially expressed by all four approaches. PI3K and pHER2 were identified as differentially expressed only in single samples. P-values for all proteins identified as differentially expressed in at least one approach are summarized in **Table 2**.

**Table 2:** Impact of sampling method on identification of proteins differentially expressed in tumors vs. normal tissues.

	Mean expression in tumors vs. expression in normal tubal epithelium	Expression in tumor sample 1* vs. expression in normal tubal epithelium	Expression in tumor sample 2* vs. expression in normal tubal epithelium	Expression in tumor sample 3* vs. expression in normal tubal epithelium
	[p-value]	[p-value]	[p-value]	[p-value]
***4E-BP1***	0.000+	0.001+	0.002+	0.001+
***EGFR***	0.000+	0.005+	0.004+	0.001+
***HER2***	0.000+	0.000+	0.002+	0.000+
***pB-Raf***	0.000−	0.026+	0.000+	0.001+
***pPTEN***	0.000+	0.008+	0.004+	0.002+
***pS6RP***	0.003+	0.005+	0.002+	0.000+
***pVEGFR***	0.006−	0.003+	0.009+	0.004+
***VEGF***	0.000+	0.000+	0.000+	0.004+
***VEGFR***	0.001−	0.002−	0.003−	0.000−
***VHL***	0.006+	0.000+	0.000+	0.000+
**pHER2**	n.s.	0.001+	0.000+	0.000+
**PI3K**	n.s.	0.026+	0.004−	0.005+
**AKT**	0.030+	n.s.	0.006+	n.s.
**pAKT**	0.018−	0.000	n.s.	n.s.
**Angiopoietin2**	0.000+	n.s.	n.s.	n.s.
**B-Raf**	0.000−	n.s.	n.s.	0.042+
**p1068EGFR**	0.000−	n.s.	n.s.	0.049+
**p1148EGFR**	0.000+	n.s.	n.s.	n.s.
**FAK**	0.000+	n.s.	n.s.	n.s.
**GSK3β**	n.s.	n.s.	n.s.	n.s.
**pGSK3β**	0.000+	0.036+	0.021+	n.s.
**Hif-1α**	0.000−	n.s.	n.s.	n.s.
**JNK/SAPK**	0.000+	n.s.	n.s.	0.021+
**mTOR**	0.000+	n.s.	n.s.	n.s.
**pmTOR**	0.000−	n.s.	n.s.	n.s.
**p4E-BP1**	n.s.	n.s.	n.s.	n.s.
**p38 MAPK**	0.000+	n.s.	n.s.	0.003+
**pp38 MAPK**	n.s.	n.s.	n.s.	n.s.
**ERK**	n.s.	n.s.	n.s.	n.s.
**pERK**	n.s.	n.s.	n.s.	n.s.
**pPRAS40**	0.000−	n.s.	n.s.	n.s.
**PTEN**	0.010+	n.s.	n.s.	n.s.
**pPDGFR**	0.003−	0.026+	n.s.	0.018+
**PDGF**	0.042−	n.s.	n.s.	n.s.
**S6RP**	0.000+	n.s.	n.s.	n.s.
**PRAS40**	0.002+	n.s.	n.s.	n.s.

* randomly selected single tumor samples per case; this analysis was repeated 3 times (tumor samples 1–3).

+ and − symbols after p-values indicate significant upregulation (+) or downregulation (−) of the respective protein in tumor vs. normal tissues.

n.s., not significant (>0.05).

*Italics* indicate the group of proteins identified as differentially expressed by all four approaches.

## Discussion

We found considerable intratumoral heterogeneity in the expression of protein biomarkers related to cell signaling pathways in high-grade serous ovarian carcinoma. All 36 proteins analyzed by reverse phase protein array (RPPA) showed a marked heterogeneity in primary ovarian cancer with a mean coefficient of variation (CV) of 25% (range 17–53%) within an individual tumor. A similar degree of variation was found among different patients with a mean CV of 21% (range 12–48%). Our results suggest that significant sampling bias can be introduced when analyzing single samples of a primary tumor for the assessment of protein biomarkers. Although all 36 candidate proteins showed considerable intratumoral heterogeneity, the overall extent of heterogeneity was different for specific proteins with p4EBP1, pp44/42 MAPK and VEGF showing highest variability and EGFR, JNK/SAPK and p38MPK showing least variability. There was no difference in the extent of heterogeneity between phosphorylated and non-phosphorylated proteins, suggesting that activated forms of cell signaling proteins can be assessed with similar reliability.

A previous study in breast cancer detected a similar degree of intratumoral heterogeneity in protein expression (mean CV 31%) [23]. In contrast, the variation of protein expression among different patients was considerably lower in ovarian cancer compared to breast cancer (mean CV 21% vs. 51%). A possible explanation is a more homogeneous patient group in our study comprising exclusively high-grade serous carcinomas, which share a common pathogenetic pathway and are therefore genetically less heterogeneous than different types of breast cancers [28]. The breast cancer study included different morphological and molecular subtypes, such as hormone receptor positive, Her2-positive and triple negative breast cancers, which may have accounted for a higher variation in protein expression between tumors from different patients.

The presence of different tumor cell clones is a potential source for intratumoral heterogeneity in protein expression. A recent study demonstrated considerable clonal intratumoral heterogeneity of ovarian cancer based on the self-renewal and tumorigenic differentiation of tumor cells derived from a single ovarian clear cell carcinoma [29]. Another study reported clonal intratumoral heterogeneity and variation between primary tumors and metastases for loss of heterozygosity of chromosomes 17q and 18q in advanced serous ovarian carcinomas [30]. Similarly, extensive clonal heterogeneity was recently demonstrated for renal cell carcinoma [31]. A comprehensive analysis of tumor cell clonality would require integrated data of DNA, RNA, and protein levels, which was beyond the scope of this study. However, a single explanation for the considerable variations in expression of cell signaling proteins found in our study is less likely. High-grade serous carcinomas are generally characterized by high proliferative and mitotic activity [28]. Although differences in tumor growth and cell proliferation may have to some degree contributed to intratumoral heterogeneity, we did not identify regional differences in tumor cell proliferation based on morphological assessment of mitoses. Another potential explanation for intratumoral heterogeneity is the cellular composition of serous ovarian cancers. However, we found no significant differences in epithelial tumor cell content and tumor stroma or inflammatory cell infiltrates between samples from the same patient.

Previous studies reported a lower degree of intratumoral variation of protein expression in ovarian cancer. Two studies from the 1990s found low variation of protein expression between different areas of 9 primary ovarian carcinomas [32] or between primary and metastatic sites of 12 epithelial ovarian cancers [33] using 2-dimensional gel electrophoresis and immunohistochemistry, respectively. However, the number of tumors in both studies was relatively small. A recent series including 123 high-grade serous ovarian carcinomas with paired ovarian tumors and omental metastases assessed expression of ten proteins by immunohistochemistry, including markers of tumor subtype, microenvironment, and cell adhesion [34]. The authors demonstrated that the majority of proteins, including commonly used diagnostic markers, such as p53, WT1, CA125, and p16 did not show significant differences in expression between primary ovarian cancer and peritoneal or omental metastases. Two markers of stromal tumor response (PDGFRB, SPARC) showed marked differential expression between primary ovarian and metastatic tumor samples [28]. However, a direct comparison of our study with previous reports is limited by the lack of uniform criteria for assessment of heterogeneity and lack of statistical measures for intratumoral variation. In addition, earlier studies have often compared primary ovarian cancers and metastatic lesions whereas our study exclusively focused on variation within a single primary cancer. A possible explanation for the higher extent of intratumoral heterogeneity in protein expression observed in this study may be the more extensive sampling of primary ovarian tumors in 5–9 different locations, as well as the higher quantitative resolution of RPPA analysis.

To further assess the variation between individual tumor samples we performed non-supervised hierarchical clustering. Although we had observed a similar extent of heterogeneity within one tumor and between tumors from different patients based on coefficient of variation, samples from the same patient clustered more closely together compared to samples from different patients. This might indicate that despite a high degree of heterogeneity in quantitative protein expression values, a ranking-based approach such as clustering may be less affected. Similarly, a comprehensive analysis of proteins in a signaling network, taking into account relative expression and ranking of individual proteins, might be less affected by heterogeneity.

Expression of cell signaling proteins is also being used as a diagnostic biomarker to distinguish tumor from associated normal tissue. We found no significant difference in cell signaling protein expression between ovarian cancer and normal serous epithelium. However, when expression values of several samples per tumor were combined, a significant difference in expression between cancer and normal epithelium was detected for the majority of proteins. This highlights the importance of multiple sampling. A practical approach in future studies may be to pool multiple samples from different regions of an individual tumor prior to analysis.

In comparison, we also assessed the physiological variation of protein expression in normal serous tubal epithelium. For technical reasons and limited availability of normal tissue samples, we could not compare different samples of the same patient. Between normal serous epithelium from different patients we observed a similar variation (mean CV 27-31%) compared to the variation between ovarian cancers from different patients (mean CV 21%).

To rule out bias due to technical variations we previously assessed the technical reproducibility of protein quantification. High reproducibility of protein measurements from independent extractions and from independent RPPA analyses was demonstrated [23] and therefore we found no reason to believe that the heterogeneity in protein expression detected in this study was attributable to technical variations.

Limitations of the current study include the number of cases and individual tumor samples (n = 88). We analyzed macroscopic heterogeneity, whereas differences on a cellular level were not assessed. An important strength is the highly homogeneous patient group comprising exclusively high-grade serous ovarian cancers known to share common pathogenetic pathways, as well as the extensive sampling of the tumors in 5–9 different areas. We analyzed different regions within each primary ovarian cancer and did not compare primary and metastatic lesions.

In conclusion, intratumoral heterogeneity can lead to sampling bias, and reliable assessment of cell signaling protein expression in ovarian cancer for assessment of prognostic or predictive biomarkers should include sampling of the primary tumor in several different locations.

## Supporting Information

Figure S1 **Comparison of tumor versus normal tissue by non-supervised hierarchical clustering of 13 tumors and 14 samples of normal serous epithelium, based on mean protein expression values per tumor.** Different patients are color-coded as indicated in the figure legend. Main clusters identified by the software are named clusters A and B. High relative expression of proteins is shown in red and low expression in green color. Grey spaces indicate missing data points.
(DOC)

Table S1 Antibodies and conditions used for protein detection.
(DOC)

Table S2 Intratumoral heterogeneity and variation between patients for the expression of 36 proteins assessed by reverse phase protein arrays (CV, coefficient of variation).
(DOC)
