# Supplementary figures and images for: Variation in Cell Signaling Protein Expression May Introduce Sampling Bias in Primary Epithelial Ovarian Cancer
Source: PLoS One. 2013 Oct 28;8(10):e77825. doi: 10.1371/journal.pone.0077825 (PMC3810127; doi:10.1371/journal.pone.0077825)

**Figure S1.**

**
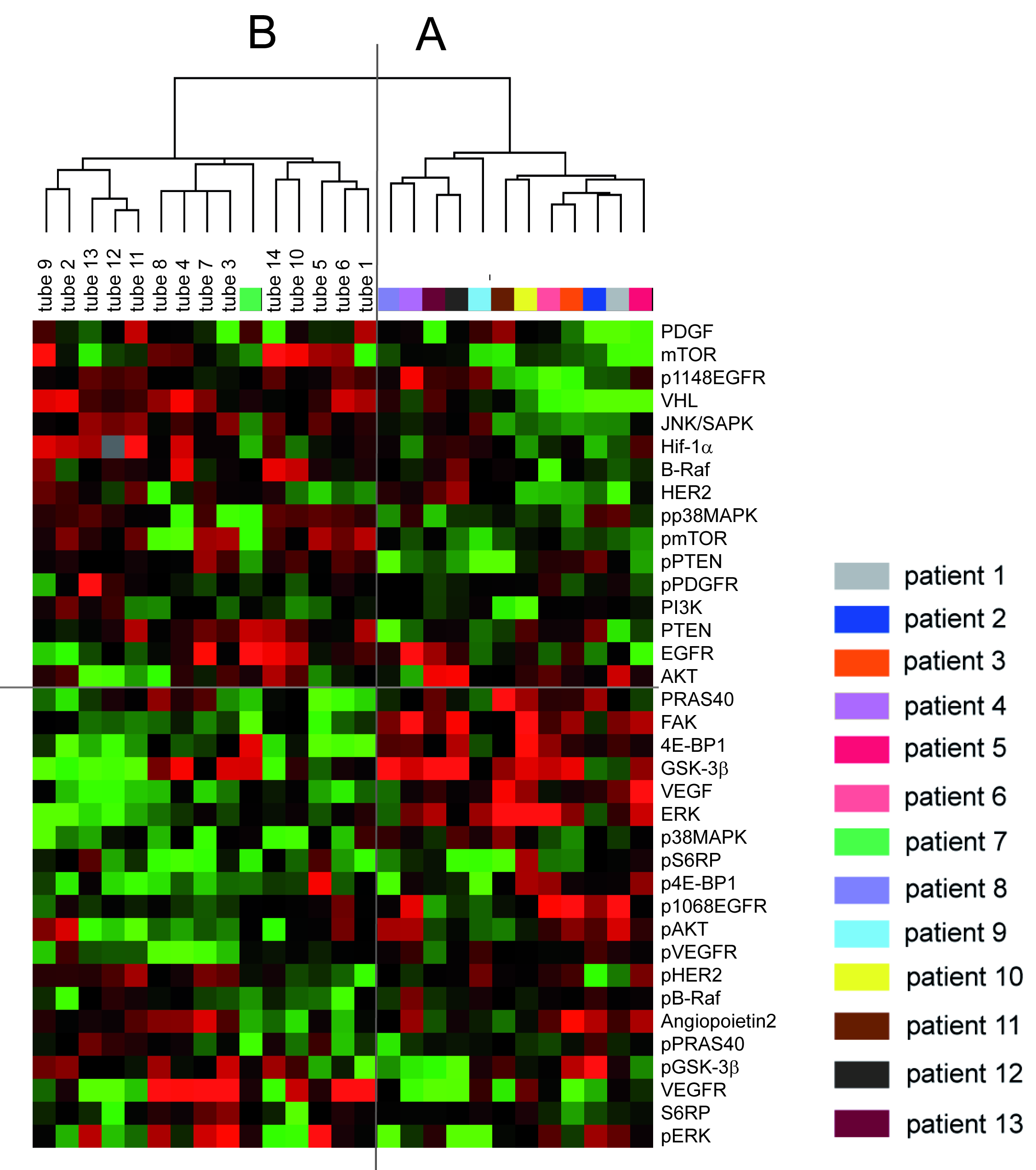
**

Supplement: Figure S1 — Comparison of tumor versus normal tissue by non-supervised hierarchical clustering of 13 tumors and 14 samples of normal serous epithelium, based on mean protein expression values per tumor. Different patients are color-coded as indicated in the figure legend. Main clusters identified by the software are named clusters A and B. High relative expression of proteins is shown in red and low expression in green color. Grey spaces indicate missing data points. (DOC) [file pone.0077825.s001.doc]
