# Supplementary material for: Variation in Cell Signaling Protein Expression May Introduce Sampling Bias in Primary Epithelial Ovarian Cancer
Source: PLoS One. 2013 Oct 28;8(10):e77825. doi: 10.1371/journal.pone.0077825 (PMC3810127; doi:10.1371/journal.pone.0077825)
Supplement: Table S1 — Antibodies and conditions used for protein detection. (DOC) [file pone.0077825.s002.doc]

Table S1.

| **Target protein** | **Molecular Weight [kDa]** | **Reference number** | **Manufacturer** | **Dilution** | **Diluted in** | **Generated in** | **Blocking solution** | **Storage tempera-ture [°C]** |
| --- | --- | --- | --- | --- | --- | --- | --- | --- |
| **4E-BP1** | 15-20 | #9644 | Cell signaling | 1:5000 | 5% BSA | rabbit | 5% MP | -20 |
| **Akt** | 60 | #9272 | Cell signaling | 1:1000 | 5% BSA | rabbit | 5% MP | -20 |
| **Angiopoietin 1** | 57 | Ab134504 | abcam | 1.500 | 5% BSA | rabbit | 5% MP | -20 |
| **Angiopoietin 2** | 56 | Ab125692 | abcam | 1:500 | 5% BSA | rabbit | 5% MP | -20 |
| **β-Aktin** | 42 | A1978, AC-15 | Sigma | 1:10000 | 5% MP | mouse | 5% MP | -20 |
| **B-Raf** | 86 | #9434 | Cell signaling | 1:500 | 5% MP | rabbit | 5% MP | -20 |
| **EGFR** | 170 | #2232 | Cell signaling | 1:2000 | 5% BSA | mouse | 5% MP | -20 |
| **FAK** | 125 | 3285 | Cell signaling | 1:3000 | 5% MP | rabbit | 5% MP | -20 |
| **GSK-3β** | 46 | #931527C10 | Cell signaling | 1:1000 | 5% MP | rabbit | 5% MP | -20 |
| **HER2** | 185 | #A0485 | Dako | 1:1000 | TBST | rabbit | 5% MP | 4 |
| **Hif-1α** | 120 | #610959 | BD | 1:1000 | 5% MP | mouse | 5% MP | -20 |
| **JNK/SAPK** | 46, 54 | #9252 | Cell signaling | 1:2000 | 5% MP | rabbit | 5% MP | -20 |
| **mTOR** | 289 | #4517 | Cell signaling | 1:2000 | 5% MP | mouse | 5% MP | -20 |
| **p38 MAPK** | 43 | #9212 | Cell signaling | 1:1000 | 5% BSA | rabbit | 5% MP | -20 |
| **p44/42 MAPK** | 42, 44 | #9102 | Cell signaling | 1:1000 | 5% BSA | rabbit | 5% MP | -20 |
| **p4E-BP1 (Thr37/46)** | 15-20 | #2855 | Cell signaling | 1:10000 | 5% MP | rabbit | 5% MP | -20 |
| **pAkt (Ser473)** | 60 | #4060 | Cell signaling | 1:1000 | 5% BSA | rabbit | 5% MP | -20 |
| **pB-Raf (Ser445)** | 86 | #2696 | Cell signaling | 1:1000 | 5% MP | rabbit | 5% MP | -20 |
| **PDGF** | 18, 29 | Ab9704 | abcam | 1:500 | 5% MP | rabbit | 5% MP | -20 |
| **p1068EGFR** | 170-180 | 369700 | invitrogen | 1:5000 | 5% BSA | rabbit | 5% MP | -20 |
| **p1148EGFR** | 170 | 4404 | Cell signaling | 1:1000 | 5% MP | rabbit | 5% MP | -20 |
| **pGSK-3β** | 46 | #9336 | Cell signaling | 1:1000 | 5% BSA | rabbit | 5% MP | -20 |
| **pHER2 (Tyr1248)** | 185 | BS4090 | BioWorld | 1:1000 | 5% MP | rabbit | 5% MP | -20 |
| **pmTOR** | 289 | #2971 | Cell signaling | 1:500 | 5% MP | rabbit | 5% MP | -20 |
| **pp38 MAPK** | 43 | #4631 | Cell signaling | 1:1000 | 5% BSA | rabbit | 5% MP | -20 |
| **pp44/42 MAPK (Thr202/204)** | 42, 44 | #9101 | Cell signaling | 1:1000 | 5% BSA | rabbit | 5% MP | -20 |
| **pPDGFR (Tyr751)** | 190 | #4549 | Cell signaling | 1:500 | 5% BSA | rabbit | 5% MP | -20 |
| **pPRAS40** | 40 | #2997 | Cell signaling | 1:1000 | 5% BSA | rabbit | 5% MP | -20 |
| **pPTEN** | 54 | #9551 | Cell signaling | 1:2000 | 5% BSA | rabbit | 5% MP | -20 |
| **pS6RP (Ser235/236)** | 32 | #4858 | Cell signaling | 1:5000 | 5% MP | rabbit | 5% MP | -20 |
| **pVEGFR-2 (Tyr1175)** | 230 | #2478 | Cell signaling | 1:1000 | 5% BSA | rabbit | 5% MP | -20 |
| **PI3K** | 85 | #4292 | Cell signaling | 1:1000 | 5% MP | rabbit | 5% MP | -20 |
| **PRAS40** | 40 | #2691 | Cell signaling | 1:1000 | 5% BSA | rabbit | 5% MP | -20 |
| **PTEN** | 54 | #9552 | Cell signaling | 1:2000 | 5% MP | rabbit | 5% MP | -20 |
| **S6RP** | 32 | #2217 | Cell signaling | 1:5000 | 5% BSA | rabbit | 5% MP | -20 |
| **VEGF** | 43 | Ab46160 | abcam | 1:1000 | 5% MP | rabbit | 5% MP | -20 |
| **VEGFR-2** | 210, 230 | #2479 | Cell signaling | 1:500 | 5% MP | rabbit | 5% MP | -20 |
| **VHL** | 24 | #2738 | Cell signaling | 1:500 | 5% MP | rabbit | 5% MP | -20 |
| **Anti-mouse-HRP** | - | NA931-1ML | GE Healthcare | 1:5000 | 5% MP | sheep | 5% MP | 4 |
| **Anti-rabbit-HRP** | - | #7074 | Cell signaling | 1:2000 | 5% MP | unknown | 5% MP | -20 |
