# Supplementary material for: Variation in Cell Signaling Protein Expression May Introduce Sampling Bias in Primary Epithelial Ovarian Cancer
Source: PLoS One. 2013 Oct 28;8(10):e77825. doi: 10.1371/journal.pone.0077825 (PMC3810127; doi:10.1371/journal.pone.0077825)
Supplement: Table S2 — Intratumoral heterogeneity and variation between patients for the expression of 36 proteins assessed by reverse phase protein arrays (CV, coefficient of variation). (DOC) [file pone.0077825.s003.doc]

Table S2.

|  | **Intratumoral heterogeneity CV [%]** | **Variation between patients CV [%]** |
| --- | --- | --- |
| Akt | 20 | 18 |
| 4E-BP1 | 25 | 18 |
| Angiopoietin2 | 27 | 22 |
| bRaf | 21 | 17 |
| EGFR | 18 | 15 |
| FAK | 24 | 23 |
| GSK-3beta | 26 | 23 |
| HER2 | 19 | 14 |
| Hif1alpha | 19 | 12 |
| JNK7SAPK | 17 | 13 |
| mTOR | 18 | 17 |
| p38MAPK | 17 | 14 |
| p44/42MAPK | 23 | 23 |
| p4E-BP1 | 37 | 37 |
| pAkt | 22 | 25 |
| PDGF | 27 | 12 |
| pEGFR(Tyr1068) | 21 | 33 |
| pEGFR(Tyr1148) | 25 | 21 |
| pGSK-3beta | 25 | 24 |
| pHER2 | 22 | 14 |
| pbRAF | 24 | 18 |
| PI3K | 24 | 12 |
| pmTOR | 22 | 18 |
| pp38MAPK | 27 | 19 |
| pp44/42MAPK | 53 | 48 |
| pPDGFR | 25 | 16 |
| pPRAS40 | 25 | 18 |
| pPTEN | 25 | 32 |
| PRAS40 | 18 | 16 |
| pS6-RP | 26 | 26 |
| PTEN | 26 | 18 |
| pVEGFR | 23 | 14 |
| S6-RP | 21 | 17 |
| VEGF | 36 | 23 |
| VEGFR | 29 | 23 |
| VHL | 25 | 16 |
| overall | 25 | 23 |
| phospho | 28 | 25 |
| non phospho | 23 | 18 |
